# Phytoremediation of cyanophos insecticide *by Plantago major* L. in water

**DOI:** 10.1186/2052-336X-12-38

**Published:** 2014-01-21

**Authors:** Ahmed Ali Romeh

**Affiliations:** 1 Plant Production Department, Faculty of Technology and Development, Zagazig University, Zagazig, Egypt

**Keywords:** Phytoremediation, *Plantago major L*, Water, Cyanophos insecticide

## Abstract

Cyanophos is commonly used in Egypt to control various agricultural and horticultural pests. It is not easily hydrolyzed and thus they are highly persistent and accumulate in various aquatic compartments such as rivers and lakes. Such issues may be solved by phytoremediation, which is the use of plants for the cleanup of pollutants. Here, we tested *Plantago major* L. to clean water polluted with cyanophos insecticide under laboratory conditions.The biosorption capacity (K_F_) of cyanophos were 76.91, 26.18 and 21.09 μg/g for dry roots, fruit (seeds with shells) and leaves of the *Plantago major* L., respectively. Viable *Plantago major* L. in water significantly reduced cyanophos by 11.0% & 94.7% during 2 hours & 9 days of exposure as compared with 0.8% & 36.9% in water without the plantain. In water with plantain, cyanophos significantly accumulated in plantain roots and leaves to reach maximum levels after two and four hours of treatment, respectively. After 1 day, the concentration of cyanophos decreased in roots and shoots until the end of testing. Three major degradation products were detected at roots and leaf samples. Here we demonstrate that *plantago major* L. removes efficiently cyanophos residue in water and has a potential activity for pesticide phytoremediation.

## Background

Cyanophos (O, O-dimethyl O-4-cyanophenyl phosphorothioate) is an organophosphorus insecticide with a commercial name of Cyanox [1]. Cyanox is commonly used in Egypt to control various agricultural and horticultural insect pests such as Hemiptera of Aphididae, Coccidae, Diaspididae, Lepidoptera, etc. in various fruits and vegetables [2]. Cyanophos used in Africa to control quelea and other granivorous species that are considered pests of cereal crops [3]. Mobile ground spraying with cyanophos control quelea, as routinely practiced in Senegal during the 1995/1996 cropping season, was found to be hazardous to the environment [1]. The toxicological effect of cyanophos is the inhibition of acetylcholine esterase activity [2]. Cyanophos is not easily hydrolyzed and highly persistent and accumulate in various aquatic compartments such as rivers and lakes [4]. Desmethyl-cyanophos, 4-Cyanophenol and desmethyl-cyanophos oxon are degradation products of cyanophos in soil [5]. All conventional methods for the removal of pesticides are found to be either uneconomical or insufficient [6]. Therefore, it becomes essential to search for effective and economical alternative method to overcome the constraints of convention methods. Biological method such as biosorption is an attractive and promising alternative which accumulate organic and inorganic matter including metal, dyes, phenols and pesticides and offers potential advantages such as low operating cost, minimization of chemical or biological sludge [7]. Several researchers reported on biosorption uptake of phenols, dyes and pesticides by biosorption [8].

Phytoremediation is an accumulation of plant-associated processes which include biotransformation, phytoaccumulation, phytoextraction, phytovolatilization, and rhizodegradation from enhanced microbial activity in plant rhizospheres [9] and plant transformation, conjugation, and sequestration are vital tools in waste management [10]. There have been several studies focused on the phytoremediation of pesticides [11,12]. Plant remediation of soils, sediments, and water is a cost-effective and resource-conservative approach for clean-up of contaminated sites [9]. Biosorption is one of the effective alternative methods for the removal of pesticides in contaminated water samples. Plants can accumulate or metabolize a variety of organic compounds, including, imidacloprid [13], triazophos [14], chlorpyrifos [12,15], methyl parathion [16], and atrazine [17].

The common broad leaved plantain (*Plantago major* L.) is a very familiar perennial weed found anywhere by roadsides, and in meadows, cultivated fields, waste areas, and canal water. The seed and husks are in fiber expanding to become highly gelatinous when soaked in water. The methanol, ethanol and aqueous extract of *Plantago major* L. contained antibacterial activity against some gram negative and positive bacteria besides a weak anti-narcotic activity [18]. The encouraging results of previous studies regarding phytoremediation gained the attention of researcher to continue studies in this field. Therefore, the objective of this work was to evaluate phytoremediation by living broadleaf plantain (*Plantago major* L.) and non- living material from plants as cleanup methods for water contaminated with the insecticide cyanophos.

## Methods

### Pesticide and plant

Cyanophos (Cyanox 50% EC) 0,0-dimethyl 0-(4-cyanophenyl) phosphorothioate was obtained from the Central Agriculture Pesticide Laboratory, Agriculture Research Center, Dokki, Gaiza, Egypt.

The common broadleaf plantain (*Plantago major* L.) used as seedlings in Phytoremediation experimentals and adult plants in biosorption assays from meadow-land in Zagazig University, Zagazig, Sharkia governorate, Egypt.

### Biosorption assays

Raw agricultural solid wastes have been used as adsorbents. These materials are available in large quantities and may have potential as adsorbents due to their physico-chemical characteristics and low-cost [19]. So, Low cost materials (leaves, roots and fruits of *Plantago major* L.) have been tested for their ability to quickly sorb cyanophos. Adults *Plantago major* L. were collected with the help of fine jet of water causing minimum damage to the roots washed thoroughly with distilled water and blotted dry. Different plant parts separated manually to leaves, roots and fruits (seeds plus shells). The plantain leaves, roots and fruits (seeds plus shells) dried naturally on laboratory benches at room temperature (28–30°C) for 5 days until crisp. Sorption was measured using 0.5 g of (powder) leaves, roots and fruits (seeds plus shells), each of the broad-leaved plantain in centrifuge tube was shaken with 10 ml of the aqueous adsorbate for four hours (equilibrium concentration). Five initial concentrations (C_B_) were used in each case, ranging 1, 5, 10, 20 and 40 μg/mL plus water blank. After centrifugation at 2000 r.p.m. for 15 minutes, the concentrations of cyanophos in the supernatant (Ce) were determined. Aliquot (4 mL) of the supernatant was analyzed. All adsorption studies were conducted at room temperature 30°C ± 2°C and three replicated were used. The amount adsorbed (μg/g) calculated [20]. Author aimed at plotting the adsorption isotherms due to which it is possible to compare the sorption capacity of cyanophos on different adsorbents (leaves, roots and fruits of *Plantago major* L.). Freundlich sorption isotherm assumes that the uptake of sorbate occurs on a heterogeneous surface by multilayer sorption and can be described by the following equation: Y = K_F_ C_e_ (n^-1^) where, K_F_ is a Freundlich constant related to the adsorption capacity (μg/g), and n^-1^ is the intensity of adsorption. The values of K_F_ and n^-1^can be determined from the intercept and slope, respectively of the linear plot of log y versus log C_e_. The empirical Freundlich isotherm often satisfactory model of experimental data [21].

### Phytoremediation assay

Whole *Plantago major* L. uptake experiment was performed in nutrient solution in Erlenmeyer flasks during test period from 2 h to 9 days. A whole *Plantago major* L. were grown in 250 ml of Hogland solution [22], containing 10 μg/ml of cyanophs in each 18 Erlenmeyer (6-periods × 3-replicates) flask 500 mL. The same number of flasks with pesticide only solution (10 μg/mL) was prepared. Three flasks were prepared as a control with a plant alone. After 2 and 4 hours, 1, 3, 6, and 9 days, three exposed and three control plants were collected. The experiment was studied at the room temperature (30 ± 2°C). Plant roots were rinsed in running tap water for 2 minutes and were blotted dry. The plants dissected into individual leaves and roots then 4 g of leaves and 2 g of roots were analyzed for the pesticide.

### Residue analysis

Water samples were extracted with methylene chloride without clean up using a continuous liquid-liquid extraction [23]. Cyanophos was extracted from the root and leaf samples with acetone or water–acetone and then extracting with petroleum ether and dichloromethane. The organic phase was separated, dried, and concentrated just to dryness [24]. The organic phase was dissolved in 5 mL of hexane then cleaned up via passing through a column prewashed with 50 ml of hexane + acetone (9: 1 v/v). The column was filled with acidic alumina (5 g) + sodium sulphate (2 mg) and was eluate with 100 mL of a mixture of hexane + acetone, 9: 1 v/v [25]. The elute was evaporated to dryness and the residue was dissolved in 1.0 mL methanol and then analyzed by high-performance liquid chromatography (HPLC) with a UV-detector at 236 nm. A C18 column was used, and the mobile phase was a mixture of methanol and water (70:30, v/v). The flow rate was 1.0 mL/min. The retention time of cyanophos was 3.46 min. The metabolite 4-cyanophenol synthesized in our laboratory by hydrolyzing cyanophos with methanolic sodium hydroxide [5] and identified by HPLC with the same condition of cyanophos. Under these conditions, the retention time of 4-cyanophenol was 1.33 min.

### Data analysis

The rate of degradation (K) and half-life (t _1/2_) was obtained from the following Equation: The rate of degradation (K) = 2.303 × slope. Half-life (t _1/2_) = 0.693/K [26].

In this study, all statistical analyses were performed with CoStat 6.311 CoHort Software. Significant differences between controls and contaminated samples were determined by the one-way ANOVA test.

Calibration curve was obtained by plotting peak areas in ‘y’ axis against concentrations of the pesticide in ‘x’ axis within the investigated range (0.18 to 12.5 μg/ml) of concentrations. Each solution was injected in triplicate. The linearity was significant with an excellent correlation coefficient of R^2^ = 0.994. The Limit of Detection (LOD) and Quantification (LOQ) of cyanophos were evaluated using the following equations: LOD = 3.3S_0_/b (3) and LOQ= 10S_0_/b (4) [27]. Where S_0_ is the standard deviation of the calibration line and b is the slope. The Limit of Detection LOD and Quantification LOQ of cyanophos in this study were found to be 0.34 μg/mL corresponding to 0.08 μg/g and 1.02 μg/mL corresponding to 0.26 μg/g, respectively. The extraction efficiency of the analytical procedure was evaluated via recovery experiments conducted in triplicate using the fortified blank *Plantago major* L. samples at two different concentrations, 0.2 and 0.5 μg/g. The average percentage recoveries obtained were between 93.1± 5.3%, 90.9± 4.5% and 88.3± 3.6% in water, leaves and roots, respectively.

## Results and discussion

### Biosorption assays

Data in Table 1 shows the biosorption capacity for cyanophos by the dry roots, fruits (seeds with shells) and leaves of *Plantago major* L. after four hours of exposure. The Experimental data of biosorption of cyanophos onto *Plantago major L.* showed that, the correlation coefficients (R^2^) for the Freundlich isotherm were 0.971, 0.997 and 0.921 for dry roots, leaves and fruit, representing a good fit of the observed data. Dry root biosorption K_F_ was much higher than that of dry fruit and dry leaves at all exposure concentrations. The amount of absorption of cyanophos can be ranked as dry roots (76.91 μg/g), dry fruits (26.18 μg/g) and dry leaves (21.09 μg/g). The equilibrium constant (n^-1^) that related to the extent or degree of biosorption was 0.896, 0.928, and 1.04 for dry roots, dry fruit, and dry leaves of *Plantago major* L., respectively. The biosorption (K_F_) of dry roots was 3.64 and 2.93 folds higher than that of dry leaves and dry fruit, while the K_F_ of dry fruit was 1.24 fold larger than that of dry leaves of *Plantago major* L. The increase of biosorption K_F_ for dry roots than dry leaves and fruits may be due to the lipid content of plant roots, in which protein, fats, nucleic acids, cellulose tissues, and other components all contain lipophilic components which appear to be more efficiency in pesticides adsorption [28]. Hetero-geneous accumulation of pesticides in different plant parts of same crop species, which could be attributed to their diverse morphological characteristics [29]. Biosorption of similar pesticides by different biomass depended on the number of sites on the biosorbent, the accessibility of the sites, the chemical state of the site (i.e., availability) and affinity between site and xenobiotic (i.e., binding strength) [30]. Also, physico-chemical parameters, such as temperature, pH of the contact solution and conditions of the reaction, e.g. continuous or batch mode, are reported to influence the adsorption results [8]. The high level of K_F_ suggested that the adsorption capacity of dry roots were high [21]. Freundlich model was more appropriate to describe the adsorption characteristics of trichloroethylene (TCE) onto Carbon nanotubes [31]. The larger Freundlich constant K_F_ showed an easy uptake of phenol from aqueous solution [32]. Agricultural fibers were more efficiency in phenol removal [33]. Furthermore, wheat ash were highly effective sorbent for herbicide MCPA [34]. Accumulation of pesticides on agricultural adsorbents is generally achieved through interactions with the hydroxyl and carboxyl groups particularly abundant in polysaccharides (cellulose and hemicelluloses) and lignin, both of which constitute about 90% of dry lignocellulosic materials [35]. The biosorption capacity (K_F_) of dry roots of *Plantago major* L. was significantly higher than that of dry leaves and dry fruits at all concentrations of imidacloprid [11]. Several researchers reported on biosorption uptake of pesticides by biosorption [8,36].

**Table 1:** **Biosorption of cyanophos by *plantago major *** **L. on a dry weight basis after 4 hours exposure**

Concentrations in water (μg/mL) ±^*^S.D.	**Concentrations in water (μg/mL) and adsorption on a dry weight basis**						**Significantly**
	**Roots**		**Leaves**		**Fruits (seeds with shells)**		
	(μg/mL) ±^*^S.D.	**(μg/g)**	(μg/mL) ±^*^S.D.	**(μg/g)**	(μg/mL) ±^*^S.D.	**(μg/g)**	
34.07 ± 1.0(a)	15.17 ± 0.67(b)	2890	11.43 ±0.5(c)	652.8	12.12 ± 0.39(c)	798.75	***
17.89 ±0.34	8.43 ± o.45(b)	946	2.23 ±0.13(c)	313.2	2.50 ±0.23(c)	384.75	***
9.10 ±0.1	4.15 ±0.13(b)	495	0.91 ±0.08(c)	182.8	0.92 ±0.03(c)	204.5	***
4.47 ± 0 .13	2.23 ± .05(b)	224	0.092 ± 0.01(c)	87.56	0.074 ± 0.002(c)	109.9	***
0.91 ±0.01	Undetected	90.0	Undetected	18.2	Undetected	22.8	***
Freundlich parameters							
^1^K_f_	76.91		21.09		26.18		
^1a^n^-1^	0.896		0.928		1.047		
^1b^R^2^	0.971		0.997		0.921		

^*^SD, standard deviation; ^1^Kf, adsorption capacity (ug/g); ^1a^n^-1^, intensity of adsorption; ^1b^R^2^, the correlation coefficient.

*P< 0.01, ***P< 0.001.

### Uptake and distribution

Table 2 show the phytoremediation potential of *Plantago major* L. to remove cyanophos insecticide from contaminated water. Viable whole plant of *Plantago major* L. in water solution significantly reduced cyanophos residues by 11.0 & 94.7% during 2 & 216 hours of exposure periods as compared with 0.8 & 36.9% in water solution without the plantain (Table 2). The half-life value (t_1/2_) of cyanophos, calculated by first-order reaction, for water planted with *Plantago major* L. and in unplanted water was found to be 1.73 and 13.63 days, respectively (Table 2). These data demonstrated that, most of the cyanophos disappearance by a *Plantago major* L. may be attributed to the uptake potential and transformation or degradation by the enzyme induction capability of the plant or by microorganisms in the plant root zone. Only one of them contributes to the reduction of a contaminant or connected them [37]. The growing cells of short-rod gram-negative bacteria that isolated from the water solution containing *Plantago major* L. was able to induce 93.34% loss of imidacloprid as a source of both carbon and nitrogen within a short period (48 hrs) compared with 31.90% in un inoculated medium [11]. The t_1/2_ value of cyanophos for Nile water was found to be 7 days [38]. Under illumination, the half-life was estimated to be 2 and 4 days on soil and silica, respectively, while it was evaluated to be 120 min in acetone solution [39]. Viable whole *Plantago major* L. in water solution reduced imidacloprid residues by 55.81–95.17%, during 1–10 days of exposure periods compared with 13.71–61.95% in water solution without *Plantago major* L. [11]. The disappearance rate constant (k_r_) is 8.0 times greater in water solution with plantain than in the cyanophos control. The areas under the curve (AUC) represent compound concentration during the period of study. AUC in the water solution with plantain was decreased than cyanophos control (Table 2).

**Table 2:** **Concentrations of cyanophs uptake during *plantago major *** **L**

**Determinations**	**2 hours**	**Days after application**							
		**0.17**	**1**	**3**	**6**	**9**	^**1**^**t**_**1/2**_**(Days**^**-1)**^	^**2**^**K**_**r**_**(Day**^**-1**^**)**	^**3**^**AUCmg l**^**1**^**(Day**^**-1**^**)**
In water solution									
μg/mL ± ^*^S.D	9.92 ±0.14a	9.85 ±0.1a	9.20 ±0.21a	8.59 ±0.12a	7.19 ±0.17a	6.31 ±0.1a	13.63	0.05	71.37
% loss	0.8	1.5	8.0	14.1	28.1	36.9			
In water solution with plantain									
μg/mL ± ^*^S.D	8.55 ±0.11c	7.70 ±0.10c	4.53 ±0.1c	2.89 ±0.03c	1.13 ±0.03	0.53 ±0.02c	1.73	0.40	20.13
% loss	11.0	23.0	54.7	71.1	88.7	94.7			
Significantly	***	***	***	***	***	***			
In plantain roots									
μg/g ± S.D	243.5 ±1.32a	113.49 ±1.56a	56.25 ±1.13a	49.21 ±1.24a	45.26 ±1.25a	42.11 ±1.35a			
In plantain leaves									
μg/g ± ^*^S.D	53.63 ±0.87b	73.0 ±1.04b	40.29 ±0.67b	36.4 ±0.86b	31.62 ±0.71b	20.00 ±1.0b			
Total uptake	297.13	186.49	96.54	85.64	76.88	30.0			
Significantly	***	***	***	***	***	***			

^1^T_1/2_, half-life; ^2^kr, disappearance rate constant; ^3^AUCs, areas under the curve represent compound concentration during the period of study; ^*^SD, standard deviation

*P± 0.01, ***P± 0.001.

**Figure 1:**
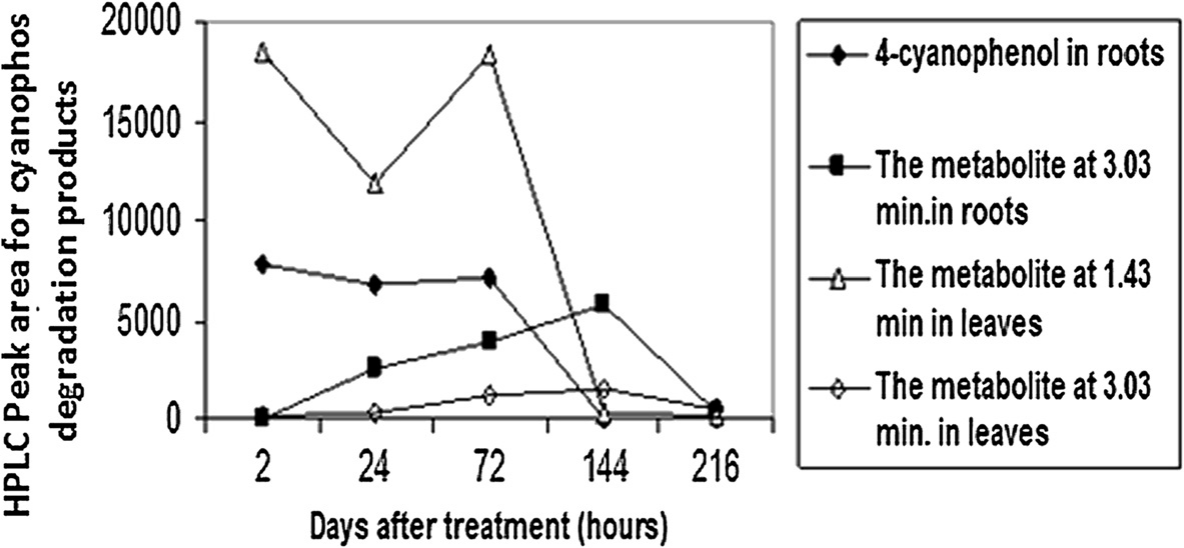
**Uptake of cyanophos degradation products in water by *Plantago major *** **L. roots and leaves.**

**Figure 2:**
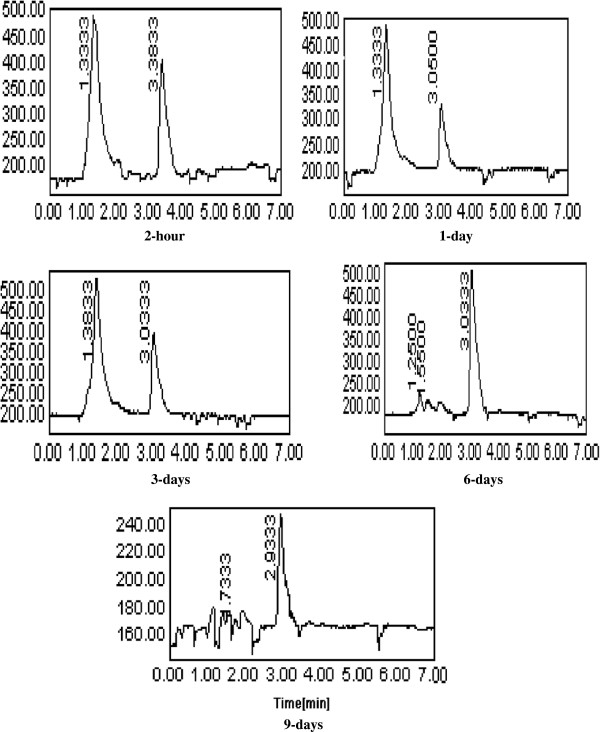
**HPLC Chromatogram of cyanophos and its degradation products in water by *Plantago major *L. roots.** Note, Retention time (R.T) of cyanophos and its degradation product 4-cyano phenol were 3.38 and 1.33 min, respectively.

**Figure 3:**
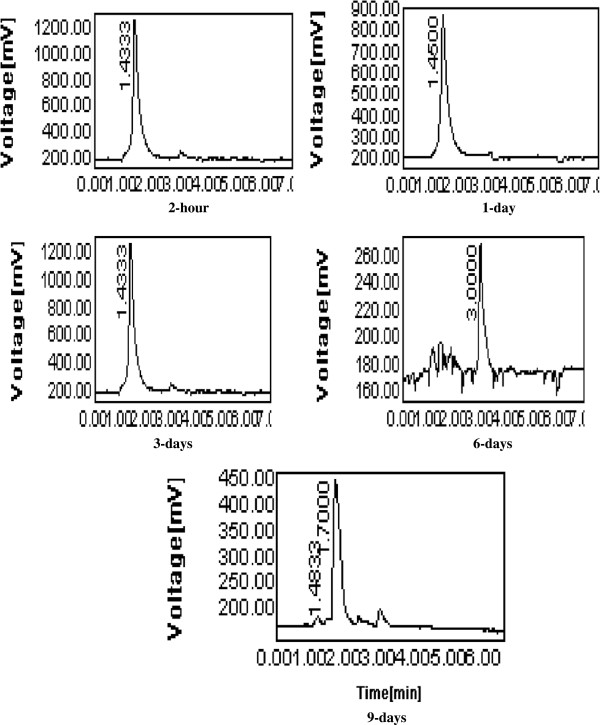
**HPLC Chromatogram of cyanophos and its products in water by *Plantago major *** **L. leaves.**

In water, cyanophos uptake and distribution by both roots and leaves of *Plantago major* L. are shown in the Table 2. Cyanophos was taken up by roots much faster than shoots. In water, Cyanophos significantly accumulated in the roots of *Plantago major* L. to reach the maximum levels after 2 hrs (243.5±1.32 μg/g). Afterwards concentration decrease gradually throughout the test. Cyanophos translocated into the leaves (53.63±0.87 μg/g) within 2 hrs and reached the maximum after 0.17 day of exposure (73.0 ±1.04 μg/g), then decreased until the end of testing (Table 2). It has been observed that roots were important in accumulating compounds due to their direct exposure of toxic chemicals with underground parts, and transporting the compounds to above-ground organs (shoots) [40]. The uptake and translocation of organic compounds are dependent on hydrophobicity (lipophilicity), solubility, polarity, molecular weight, plant species and environmental factors [41]. Lipophilicity is the most important property of a chemical in determining its movement into and within a plant and is related to the n-octanol/water partition coefficient (K_ow_) value. For uptake, log K_ow_ must be typically between 0.5 and 3.0. Compounds with larger log K_ow_ are hydrophobic and may adsorb strongly onto roots. With smaller log K_ow_ are too hydrophilic to pass through cell membrane [42]. Cyanophos is a moderately hydrophobic compound (log K_ow_ 2.65) and is likely to partially adsorb onto roots or be taken up by roots and move across cell membranes to reach the aboveground portion of plants. Foliar uptake directly into the aboveground portion of plants is an important route compared with root uptake, especially for volatile and semi-volatile compounds [43]. However, the volatility of cyanophos was found to be substantially high, V.p. 105 at 20°C [2] therefore, cyanophos residue accumulated in the roots and moved to the aerial part of the plant material. Also, potential uptakes of organic contamination are influenced by evapotranspiration [44]. Pesticide remediation is enhanced in emergent and floating vegetation by high transpiration rates and lipids associated with plant cuticles [44], therefore higher transpiration rates of emergent vegetation could be expected to increase volatilization of pesticide metabolites resulting in greater remediation capabilities. Most of the transport of pesticides occurs in aquatic plants via rhizomes. Once moved inside the plants, pesticides are acropetally distributed from the roots primarily into the leaves and lost via diffusion if volatile [45]. The rooted portion of *Acorus. gramineus* plays an import role in the total pesticide-uptake due to the relatively high weight-based accumulation of pesticides and its contribution to organ biomass proportion [46]. Among the organophosphorus pesticide, malathion was an efficient accumulator (81%) in the roots, followed by fenitrothion (76%), diazinon (65%), and parathion (64%), respectively. In water solution with *Plantago major* L., imidacloprid significantly accumulated in plantain roots, leaves and fruits to reach the maximum levels after 6, 1 and 3 days of treatment, respectively. The maximum levels were 15.74, 37.21, and 5.74 μg/g, respectively. These values were decreased to 6.95, 1.46, and 0.12 μg/g after 10 days of treatment [11]. Plants can accumulate or metabolize a variety of organic compounds, including, imidacloprid [13], triazophos [14], chlorpyrifos [12,15], methyl parathion [16] and atrazine [17,47]. Several grass species were utilized to create grass waterways and buffer strips to contain polluted surface waters [48-50]. The disappearance of cyanophos was coupled with the appearance of the metabolites in the roots and the leaves of *Plantago major* L. Three major degradation products were detected at roots and leaf samples (Figures 1, 2 and 3). Two major metabolites 4-cyanophenol and product with retention time (R.T.) of 1.43 minutes were detected in roots and leaves, respectively. One other metabolite was detected in both roots and leaves at a R.T. of 3.05 minutes (Figures 1 and 2). In the water solution, the degradation products 4-cyanophenol and the product at a R.T. of 1.43 minutes were detected in the roots and the leaves after two hours, respectively. After1 day, the product at a R.T. of 1.43 minutes was decreased gradually in leaves until the end of sampling while the 4-cyanophenol was no detectable in roots at 6 days (Figures 1 and 2). The metabolite at a R.T. of 3.05 minutes was detected in both roots and leaves after 1 day and 2 hours, respectively. The metabolite at a R.T. of 3.05 minutes increased gradually to 6 days then decreased throughout the 9 days of exposure (Figure 1). These data indicated that *Plantago major* L., degrade cyanophos enzymatically in water. Cyanophos was taken up rapidly and metabolized by the root of bean in hydroponic solution [5]. After 9 days, 24% remained as unchanged and 24% as desmethyl-cyanophos via demethylation. The important metabolite 4-cyanophenol a probably occurs principally by the hydrolysis of Oxon in plants. The same author found that when cyanophos was applid to bean leaves cyanophos oxon and desmethyl-cyanophos oxon were also detected. The aerobic metabolism of cyanophos in water-sediment systems undergoes the cleavage of the P–O-methyl and P–O-aryl bonds together with the oxidation of the P=S to the oxon group and the hydrolysis of the cyanophos moiety [51]. Photo-oxidation reaction leading to the formation of the oxon derivative and the scission of the P–O bond generating 4-cyanophenol, as a major product are reported to be the main observed processes [39]. Wheat plants enhanced uptake/degradation of methyl parathion, p-nitrophenol and hydroquinone in unsterilized soil by 64.85%, 94.7% and 55.8% respectively. Methyl parathion hydrolyzes to p-nitrophenol, which is further metabolized to hydroquinone with nitrite release The enzyme p-nitrophenol 4-hydroxylase is active as evidenced by release of nitrite by leaf and root extracts and also by the appearance of hydroquinone in the reaction mixture [16]. Data also in Figures 2 and 3 clear role *Plantago major* L. in phytoremediation of cyanophos and degradation products in water.

## Conclusions

The use of plants to detoxify contaminated water is a potentially cost-effective alternative to traditional remediation technologies. From the results of this study, it can be ended that the existence of plants increased the removal t_1/2_ of cyanophos in water system. *Plantago major* L. is able to take up cyanophos from water by roots as well as by leaves, so *Plantago major* L. may be used for phytoremediation of water contaminated with cyanophos insecticide.

## Competing interests

The authors declare that they have no competing interests.

## Authors’ contributions

This search individually not shared by one of the researchers. I have made substantial contributions to conception and design, or acquisition of data, or analysis and interpretation of data; 2) I have been involved in drafting the manuscript or revising it critically for important intellectual content; and 3) I have given final approval of the version to be published.
